# Postoperative Delirium in Older Adults Undergoing Noncardiac Surgery

**DOI:** 10.1001/jamanetworkopen.2025.19467

**Published:** 2025-07-08

**Authors:** Heather L. Lander, Andrew W. Dick, Karen E. Joynt Maddox, Mark A. Oldham, Lee A. Fleisher, Michael Mazzeffi, Stewart J. Lustik, Jingjing Shang, Patricia W. Stone, Marjorie S. Gloff, Jacob Nadler, Isaac Wu, Raymond Zollo, Laurent G. Glance

**Affiliations:** 1 Department of Anesthesiology and Perioperative Medicine, University of Rochester School of Medicine, Rochester, New York; 2 RAND Health, RAND, Boston, Massachusetts; 3 Department of Medicine, Washington University in St Louis, St Louis, Missouri; 4 Center for Advancing Health Services, Policy & Economics Research, Washington University in St Louis, St Louis, Missouri; 5 Department of Psychiatry, University of Rochester School of Medicine, Rochester, New York; 6 Department of Anesthesiology, University of Pennsylvania, Philadelphia; 7 Leonard Davis Institute of Health Economics, University of Pennsylvania, Philadelphia; 8 Department of Anesthesiology, University of Virginia, Charlottesville; 9 Columbia University School of Nursing, New York, New York; 10 Department of Public Health Sciences, University of Rochester School of Medicine, Rochester, New York

## Abstract

**Importance:**

Understanding the association of postoperative delirium with adverse outcomes and the hospital-level variation of postoperative delirium is important for efforts to improve perioperative brain health.

**Objective:**

To examine (1) the association of postoperative delirium with 30-day mortality and complications and (2) hospital-level variation in postoperative delirium.

**Design, Setting, and Participants:**

This retrospective cohort study examined hospitalizations among patients aged 65 years and older who underwent noncardiac surgery in US hospitals between January 1, 2017, and December 31, 2020. Data were analyzed between August 28, 2024, and April 10, 2025.

**Exposure:**

Postoperative delirium.

**Main Outcomes and Measures:**

The association of the composite of death and major complications with postoperative delirium was examined using multivariable logistic regression. Variability in the hospital incidence of postoperative delirium was evaluated using multilevel logistic regression analysis.

**Results:**

Among 5 530 054 inpatient admissions for major noncardiac surgery in 3169 hospitals, the mean (SD) patient age was 74.7 (7.0) years, and 3 161 054 admissions (57.2%) were of female patients. The incidence of postoperative delirium was 3.6% (197 921 admissions). Compared with patients without postoperative delirium, patients with postoperative delirium were more likely to experience death or major complications (adjusted OR [aOR], 3.47; 95% CI, 3.41-3.53; *P* < .001), 30-day mortality (aOR, 2.77; 95% CI, 2.71-2.83; *P* < .001), and nonhome discharges (aOR, 3.96; 95% CI, 3.88-4.04; *P* < .001). Controlling for patient characteristics, the odds of postoperative delirium were higher for patients undergoing surgery in hospitals with a higher rate of postoperative delirium compared with hospitals with lower rates of postoperative delirium (median OR, 1.53; 95% CI, 1.50-1.56).

**Conclusions and Relevance:**

In this national retrospective cohort study of more than 5.5 million hospitalizations, older individuals undergoing major noncardiac surgery who experienced postoperative delirium had 3.5-fold higher odds of death or major complications, 2.8-fold higher odds of death, and 4.0-fold higher odds of nonhome discharge. There was substantial variation in the hospital rate of postoperative delirium after accounting for patient risk, which suggests that this complication may be an appropriate target for hospital efforts to improve perioperative brain health, provided that delirium screening and coding accuracy are improved.

## Key Points

**Question:**

What is the association of postoperative delirium with adverse outcomes, and how much variation exists in hospital rates of postoperative delirium?

**Findings:**

In this cohort study of 5 530 054 older individuals undergoing major noncardiac surgery, those who experienced postoperative delirium had 3.5-fold higher odds of death or major complications, 2.8-fold higher odds of death, and 4.0-fold higher odds of nonhome discharge. The odds of postoperative delirium were 1.5-fold higher for patients undergoing surgery in hospitals with a higher rate of postoperative delirium compared with hospitals with lower rates of postoperative delirium.

**Meaning:**

These findings suggest that postoperative delirium may be an appropriate target for hospital efforts to improve perioperative brain health provided that delirium screening and coding accuracy are improved.

## Teaser

This cohort study examines hospital-level variation in postoperative delirium and associations of postoperative delirium with mortality and major complications among older adults undergoing noncardiac surgery.

## Introduction

Delirium is an acute, often reversible, change in cognition associated with inattention, impaired consciousness, and disorganized thinking.^1,2^ One major cause of delirium is surgery. The incidence of postoperative delirium among older individuals undergoing surgery varies widely, with reported rates ranging between 0.7% and 50%.^3,4,5,6,7,8,9,10,11,12,13,14^ Delirium is associated with an increased risk of death, institutionalization, long-term cognitive decline, and dementia.^15,16,17,18,19,20,21,22^ Postoperative delirium in older adults is estimated to lead to between $26 and $42 billion in health care costs per year.^21^ While delirium was previously considered benign and self-limited, views have shifted in recent years. Indeed, postoperative delirium has been described in a best practice statement from the American Geriatrics Society as acute brain failure, analogous to acute heart failure.^3,23,24^ The American Geriatrics Society argues that, like acute heart failure, acute brain failure should be treated as a medical emergency requiring rapid diagnosis and treatment.^25^ With the increasing recognition that delirium may lead to dementia, postoperative delirium has become a public health priority.^20,26,27,28,29^

The cause of delirium is multifactorial and includes predisposing factors such as underlying cognitive dysfunction, advanced age, and multimorbidity, as well as potentially modifiable factors such as polypharmacy and the use of psychotropic medications, infection, kidney insufficiency, hypoxia, hypercarbia, dehydration, immobilization, poor vision or hearing, and sleep deprivation.^3,23^ A recent American College of Surgeons (ACS) geriatric pilot study using data on 20 212 older adults undergoing inpatient surgical procedures at 30 hospitals reported that rates of postoperative delirium varied 8.5-fold across hospitals.^30^ This variability, along with a growing understanding of the modifiable factors noted previously, suggests that postoperative delirium is an appropriate target for surgical quality improvement.^30^ To this end, preventing postoperative delirium is the focus of guidelines from the American Geriatrics Society and the ACS. Recommendations include early and frequent assessment, and both pharmacologic measures (regional anesthesia, optimization of pain control with nonopioid medications, and avoidance of sedative hypnotics) and nonpharmacologic measures (sensory enhancement [ie, glasses or hearing aids], early ambulation, sleep enhancement, and nutrition).^3^

While substantial progress has been made in conceptualizing postoperative delirium, there is, to our knowledge, no nationally representative data examining the scope of the problem or its association with clinical outcomes. Our first aim was thus to examine the association of postoperative delirium with 30-day mortality and complications, 30-day mortality, and nonhome discharges in older adults undergoing inpatient noncardiac surgery using population-based data on more than 5 million older US residents using Medicare claims data. Building on the prior work of the ACS geriatric pilot study,^30,31^ our second aim was to examine hospital-level variability in the risk-adjusted incidence of postoperative delirium. Understanding hospital-level variation in postoperative delirium, and the association of postoperative delirium with other important postoperative outcomes, may help inform further development of evidence-based, comprehensive prevention and treatment programs to improve health care outcomes in older surgical patients.

## Methods

The institutional review board of the University of Rochester School of Medicine and Dentistry reviewed the proposal for this cohort study and determined that it met federal and university criteria for exemption because it consisted of secondary research on preexisting data and therefore waived the requirement for informed consent. The Strengthening the Reporting of Observational Studies in Epidemiology (STROBE) reporting guideline^32^ was used to guide the reporting of this study. Data were analyzed between August 28, 2024, and April 10, 2025.

### Data Source

This retrospective cohort analysis was based on data from the 100% Medicare Provider Analysis and Review File and the Master Beneficiary Summary File between January 1, 2017, and December 31, 2020. These files include beneficiary demographic information (age, sex, and self-reported race and ethnicity); *International Statistical Classification of Diseases, Tenth Revision, Clinical Modification (ICD-10-CM) *diagnosis and procedure codes; present-on-admission codes to distinguish between preexisting conditions and complications; source of admission (community, hospital transfer, skilled nursing facility, or nursing home); urgency of admission (elective, urgent, or emergent); payer status (dual enrollment in Medicare and Medicaid as a proxy for low socioeconomic status); and information on discharge destination, death, and hospital identifiers. Race and ethnicity categories included American Indian and Alaska Native, Asian or Pacific Islander, Black, Hispanic, White, and other race or ethnicity (no other information is available on the groups included in other race and ethnicity); race and ethnicity were included to serve as a proxy for unmeasured comorbidity burden. These files were merged with data from the Centers for Medicare & Medicaid Services Impact Files, which include information on hospital characteristics such as the hospital disproportionate share percentage, resident-to-bed ratio, average daily census, rurality, and geographic region.

### Study Population

**Figure 1.**
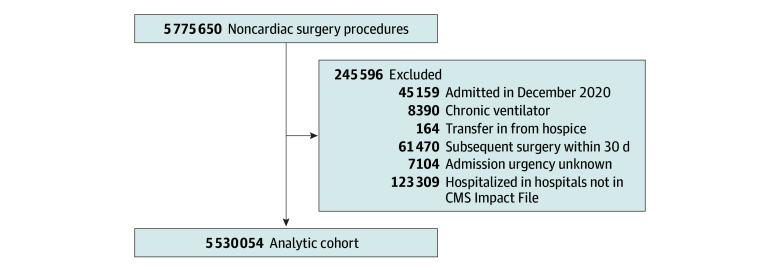
Flow Diagram Describing Selection of Cases in the Analytic Cohort CMS indicates Centers for Medicare & Medicaid Services.

We identified 5 775 650 hospitalizations in patients aged 65 years and older for noncardiac surgery between January 1, 2017, and December 31, 2020 (Figure 1). These surgical procedures were identified using codes listed in the 2021 Centers for Disease Control and Prevention National Healthcare Safety Network *International Statistical Classification of Diseases and Related Health Problems, Tenth Revision (ICD-10) *operative procedure code mappings.^33^ We excluded patients admitted in December 2020 to have a uniform 30-day look-forward period for 30-day mortality (45 159 individuals).

### Key Variables

The primary outcome was the composite of death and major complications (30-day mortality, acute myocardial infarction, acute ischemic stroke, and congestive heart failure). Secondary outcomes were 30-day mortality and nonhome discharge (death or discharge to a skilled nursing facility, long-term care hospital, or hospital transfer).

The exposure of interest was postoperative delirium, defined using *ICD-10* codes for delirium and encephalopathy (eTable 1 in Supplement 1).^34,35^ We included toxic, metabolic, and other causes of encephalopathy because delirium is a manifestation of acute encephalopathy and frequently coded using codes for encephalopathy (eTable 1 in Supplement 1). Notably, there is a strong financial incentive to code changes in neurocognitive states in claims data using encephalopathy instead of delirium codes; encephalopathy is classified as a major complication or comorbidity while delirium is classified as a comorbidity only in the Medicare severity diagnosis-related group classification for hospital reimbursement.^31^

### Statistical Analysis

Our first aim was to examine the association of the composite of death and major complications with postoperative delirium. We estimated a logistic regression model that controlled for patient demographics (age, sex, race, and ethnicity); payer status (Medicare and dual enrollment in Medicare and Medicaid); urgency (elective, urgent, and emergent); admission source (community, hospital transfer, skilled nursing facility or nursing home, or other); frailty (wheelchair use, supplemental oxygen, malnutrition, urinary incontinence, fecal incontinence, gait disturbance, history of falls, or dependence on caregiver); COVID-19 infection on admission; Elixhauser comorbidities; percutaneous or laparoscopic vs open surgical approach; surgical procedure; and day of the week, month, and year of admission. The present-on-admission codes, which differentiate diagnoses that were present on admission from complications that occur during hospital admission, were used to identify outcomes and comorbidities. Surgery procedure categories were specified as separate indicator variables using the 2021 Centers for Disease Control and Prevention National Healthcare Safety Network *ICD-10* operative procedure code mappings.^33^ We separately specified the components of the Hospital Frailty Score^36^ that were not included in the Elixhauser Comorbidity Algorithm (eTable 1 in Supplement 1). We also performed secondary analyses examining the association of postoperative delirium with (1) 30-day mortality and (2) nonhome discharges. We started by first estimating sequential nonhierarchical models in which we adjusted for (1) age and procedure; (2) age, procedure, and patient characteristics; and (3) age, procedure, and patient and hospital characteristics. We used the same approach for the other key outcomes. In addition, we performed 2 post hoc analyses, which were requested during peer review of this article. In the first subgroup analysis, we excluded patients admitted in 2020 to limit the analytic cohort to patients not admitted during the COVID-19 pandemic. In the second subgroup analysis, we examined the interaction of sex with postoperative delirium.

Our second aim was to examine hospital variability in the risk-adjusted rate of postoperative delirium. We estimated a multilevel logistic regression model in which hospitals were specified as a random effect using 2019 data. We controlled for the same covariates described previously. We used the median odds ratio (OR) to quantify the variation in the hospital odds of postoperative delirium.^37^ The median OR is the median increase in odds of postoperative delirium when a patient undergoes surgery at a hospital with a higher risk of postoperative delirium vs a hospital with a lower risk of postoperative delirium when the hospital pairs are selected at random after controlling for patient risk and using all possible combinations of hospitals.^37^

We also conducted an additional analysis to quantify the hospital-level variability in the risk-adjusted odds of postoperative delirium. First, we identified hospitals with statistically higher or lower rates of postoperative delirium as those hospitals whose risk-adjusted ORs, based on the random-intercept model, were significantly greater or less than 1, respectively. We then examined whether patients undergoing surgery in 2020 in hospitals identified as having significantly higher or lower risk-adjusted rates of postoperative delirium in 2019 would be more or less likely to experience postoperative delirium. Using 2020 data, we specified a multivariable nonhierarchical logistic regression model with postoperative delirium as the outcome of interest and the hospital incidence of risk-adjusted postoperative delirium based on the 2019 data (specified as a categorical variable: significantly higher rate, significantly lower rate, or average rate) as the exposure of interest. We controlled for the same list of patient characteristics described previously.

We then conducted an exploratory analysis examining the association of postoperative delirium and the estimated mortality risk. We first specified a multivariable logistic regression model with 30-day mortality as the outcome using the full analytic cohort. We then used this model to estimate the risk of death for each patient. We then re-estimated a separate model in which the outcome was postoperative delirium and the exposure of interest was the risk of death specified as a categorical variable.

Data management and statistical analyses were performed using STATA SE/MP version 18.0 (Stata Corp). All statistical tests were 2-tailed, and *P* values less than .05 were considered significant. We used cluster robust variance estimators in the nonhierarchical models to account for the clustering of observations within hospitals. Because data were missing on approximately 0.1% of cases, we excluded cases with missing data from the analytic dataset and performed complete case analysis.

## Results

### Patient Characteristics

This study was based on data from 5 530 054 admissions from 3169 hospitals (Table). The mean (SD) patient age was 74.7 (7.0) years, and 3 161 054 admissions (57.2%) were of female patients. Among these admissions, 22 105 (0.4%) were of American Indian or Alaska Native patients, 96 038 (1.7%) were of Asian or Pacific Islander patients, 393 614 (7.1%) were of Black patients, 328 134 (5.9%) were of Hispanic patients, 4 567 414 (82.6%) were of White patients, and 34 203 (0.6%) were of patients classified as other race or ethnicity. Among these admissions, 197 921 (3.6%) were in patients who experienced postoperative delirium and 5 332 133 (96.4%) were in patients who did not (Table). Patients with postoperative delirium were slightly older (mean [SD] age, 78.3 [8.0] years vs 74.5 [6.9] years), more likely to be male (91 148 individuals [46.1%] vs 2 277 852 individuals [42.7%]), less likely to be admitted electively (86 931 individuals [43.9%] vs 3 887 334 individuals [72.9%]), and less likely to be admitted from the community (178 026 individuals [90.0%] vs 5 139 166 individuals [96.4%]). Patients with postoperative delirium were more likely to have comorbidities, including malnutrition (35 083 individuals [17.7%] vs 231 465 individuals [4.3%]), cerebrovascular disease (10 626 individuals [5.4%] vs 139 392 individuals [2.6%]), heart failure (41 320 individuals [20.9%] vs 434 386 individuals [8.2%]), dementia (34 687 individuals [17.5%] vs 230 213 individuals [4.3%]), paralysis (8660 individuals [4.4%] vs 98 350 individuals [1.8%]), moderate kidney failure (35 840 individuals [18.1%] vs 549 976 individuals [10.3%]), and severe kidney failure (12 395 individuals [6.3%] vs 121 421 individuals [2.3%]) than patients without postoperative delirium (Table). Hospital characteristics are shown in eTable 2 in Supplement 1.

**Table.** Patient Characteristics

Characteristics	Patients, No. (%)		
	Total (N = 5 530 054)	No postoperative delirium (n = 5 332 133)	Postoperative delirium (n = 197 921)
Age, mean (SD), y	74.7 (7.0)	74.5 (6.9)	78.3 (8.0)
Sex			
Male	2 369 000 (42.8)	2 277 852 (42.7)	91 148 (46.1)
Female	3 161 054 (57.2)	3 054 281 (57.3)	106 773 (54)
Race and ethnicity			
American Indian or Alaska Native	22 105 (0.4)	21 185 (0.4)	920 (0.5)
Asian or Pacific Islander	96 038 (1.7)	92 900 (1.7)	3138 (1.6)
Black	393 614 (7.1)	378 555 (7.1)	15 059 (7.6)
Hispanic	328 134 (5.9)	318 797 (6)	9337 (4.7)
White	4 567 414 (82.6)	4 400 755 (82.5)	166 659 (84.2)
Other^a^	34 203 (0.6)	33 002 (0.6)	1201 (0.6)
Dual-eligible	692 517 (12.5)	656 754 (12.3)	35 763 (18.1)
Admission urgency			
Elective	3 974 265 (71.9)	3 887 334 (72.9)	86 931 (43.9)
Emergent	1 168 647 (21.1)	1 080 689 (20.3)	87 958 (44.4)
Urgent	387 142 (7.0)	364 110 (6.8)	23 032 (11.6)
Admission source			
Community	5 317 192 (96.2)	5 139 166 (96.4)	178 026 (90.0)
Hospital transfer	152 200 (2.8)	138 299 (2.6)	13 901 (7.0)
Skilled nursing facility or nursing home	36 105 (0.7)	32 035 (0.6)	4070 (2.1)
Other	24 557 (0.4)	22 633 (0.4)	1924 (1.0)
Functional status and frailty			
Wheelchair use	18 267 (0.3)	16 884 (0.3)	1383 (0.7)
Supplemental oxygen	83 213 (1.5)	78 133 (1.5)	5080 (2.6)
Malnutrition	266 548 (4.8)	231 465 (4.3)	35 083 (17.7)
Urinary incontinence	67 777 (1.2)	63 093 (1.2)	4684 (2.4)
Fecal incontinence	12 876 (0.2)	11 926 (0.2)	950 (0.5)
Gait disturbance	34 577 (0.6)	32 527 (0.6)	2050 (1.0)
History of falls	8250 (0.2)	7428 (0.1)	822 (0.4)
Dependent on caregiver	16 098 (0.3)	14 990 (0.3)	1108 (0.6)
Previous myocardial infarction			
None	5 510 393 (99.6)	5 314 748 (99.7)	195 645 (98.9)
Prior ST segment elevation myocardial infarction	1456 (0.03)	1236 (0.02)	220 (0.11)
Prior non–ST segment elevation myocardial infarction	8785 (0.2)	7884 (0.2)	901 (0.5)
Prior other myocardial infarction	9420 (0.2)	8265 (0.2)	1155 (0.6)
COVID-19	3028 (0.1)	2710 (0.1)	318 (0.2)
Elixhauser comorbidities			
AIDS	6644 (0.1)	6384 (0.1)	260 (0.1)
Alcohol abuse	79 098 (1.4)	70 676 (1.3)	8422 (4.3)
Deficiency anemia	576 656 (10.4)	542 556 (10.2)	34 100 (17.2)
Autoimmune conditions	248 612 (4.5)	238 676 (4.5)	9936 (5.0)
Lymphoma	31 747 (0.6)	29 784 (0.6)	1963 (1.0)
Leukemia	25 848 (0.5)	24 405 (0.5)	1443 (0.7)
Metastatic cancer	186 685 (3.4)	176 059 (3.3)	10 626 (5.4)
Solid tumor	135 233 (2.5)	128 225 (2.4)	7008 (3.5)
Cerebrovascular disease	150 159 (2.7)	139 392 (2.6)	10 767 (5.4)
Heart failure	475 706 (8.6)	434 386 (8.2)	41 320 (20.9)
Coagulopathy	158 170 (2.9)	145 683 (2.7)	12 487 (6.3)
Dementia	264 900 (4.8)	230 213 (4.3)	34 687 (17.5)
Depression	705 105 (12.8)	670 445 (12.6)	34 660 (17.5)
Diabetes without chronic complications	754 537 (13.6)	734 774 (13.8)	19 763 (10.0)
Diabetes with chronic complications	659 436 (11.9)	617 860 (11.6)	41 576 (21.0)
Drug abuse	37 526 (0.7)	34 549 (0.7)	2977 (1.5)
Hypertension, complicated	1 004 875 (18.2)	935 297 (17.5)	69 578 (35.2)
Hypertension, uncomplicated	3 062 880 (55.4)	2 976 100 (55.8)	86 780 (43.9)
Liver disease, mild	160 285 (2.9)	153 587 (2.9)	6698 (3.4)
Liver disease and failure, moderate to severe	19 293 (0.4)	17 564 (0.3)	1729 (0.9)
Chronic pulmonary disease	1 061 823 (19.2)	1 011 702 (19)	50 121 (25.3)
Neurologic disorder, movement disorder	189 524 (3.4)	177 883 (3.3)	11 641 (5.9)
Seizures and epilepsy	75 715 (1.4)	70 328 (1.3)	5387 (2.7)
Obesity	1 094 989 (19.8)	1 062 889 (19.9)	32 100 (16.2)
Paralysis	107 010 (1.9)	98 350 (1.8)	8660 (4.4)
Peripheral vascular disease	373 370 (6.8)	352 388 (6.6)	20 982 (10.6)
Psychoses	86 081 (1.6)	80 538 (1.5)	5543 (2.8)
Pulmonary circulation disease	112 088 (2.0)	102 662 (1.9)	9426 (4.8)
Kidney failure, moderate	585 816 (10.6)	549 976 (10.3)	35 840 (18.1)
Kidney failure, severe	133 816 (2.4)	121 421 (2.3)	12 395 (6.3)
Hypothyroidism	1 040 673 (18.8)	1 000 782 (18.8)	39 891 (20.2)
Thyroid, other disorders	66 541 (1.2)	64 045 (1.2)	2496 (1.3)
Peptic ulcer disease with bleeding	44 936 (0.8)	41 788 (0.8)	3148 (1.6)
Valvular disease	334 958 (6.1)	315 913 (5.9)	19 045 (9.6)
Procedural approach			
Open	4 557 707 (82.4)	4 385 168 (82.2)	172 539 (87.2)
Percutaneous or laparoscopic	972 347 (17.6)	946 965 (17.8)	25 382 (12.8)
Procedure			
Abdominal aortic aneurysm repair	6096 (0.1)	5272 (0.1)	824 (0.4)
Bile duct, liver, or pancreatic surgery	66 376 (1.2)	62 142 (1.2)	4234 (2.1)
Carotid endarterectomy	178 896 (3.2)	175 876 (3.3)	3020 (1.5)
Colon surgery	446 727 (8.1)	421 808 (7.9)	24 919 (12.6)
Spinal fusion	597 047 (10.8)	569 960 (10.7)	27 087 (13.7)
Gastric surgery	102 494 (1.9)	97 527 (1.8)	4967 (2.5)
Hysterectomy	60 689 (1.1)	59 491 (1.1)	1198 (0.6)
Rectal surgery	37 348 (0.7)	36 039 (0.7)	1309 (0.7)
Arthroplasty of knee	1 377 613 (24.9)	1 356 303 (25.4)	21 310 (10.8)
Arthroplasty of hip	1 261 388 (22.8)	1 209 907 (22.7)	51 481 (26.0)
Laminectomy	144 358 (2.6)	138 723 (2.6)	5635 (2.9)
Neck surgery	19 618 (0.4)	18 968 (0.4)	650 (0.3)
Kidney surgery	95 180 (1.7)	92 352 (1.7)	2828 (1.4)
Ovarian surgery	15 344 (0.3)	14 887 (0.3)	457 (0.2)
Prostate surgery	81 169 (1.5)	80 468 (1.5)	701 (0.4)
Peripheral vascular bypass surgery	84 430 (1.5)	80 151 (1.5)	4279 (2.2)
Small bowel surgery	182 225 (3.3)	169 370 (3.2)	12 855 (6.5)
Spleen surgery	8181 (0.2)	7593 (0.1)	588 (0.3)
Thoracic surgery	243 228 (4.4)	233 111 (4.4)	10 117 (5.1)
Thyroid and/or parathyroid surgery	19 651 (0.4)	19 236 (0.4)	415 (0.2)
Exploratory laparotomy	159 475 (2.9)	150 399 (2.8)	9076 (4.6)
Gallbladder surgery	342 521 (6.2)	332 550 (6.2)	9971 (5.0)
Outcomes			
Death or major complication	194 894 (3.5)	156 309 (2.9)	38 585 (19.5)
Death	137 543 (2.5)	111 395 (2.1)	26 148 (13.2)
Nonhome discharge	1 461 928 (26.4)	1 325 980 (24.9)	135 948 (68.7)

^a^ No other information is available on the race and ethnicity of individuals included in the category other.

### Unadjusted Outcomes

**Figure 2.**
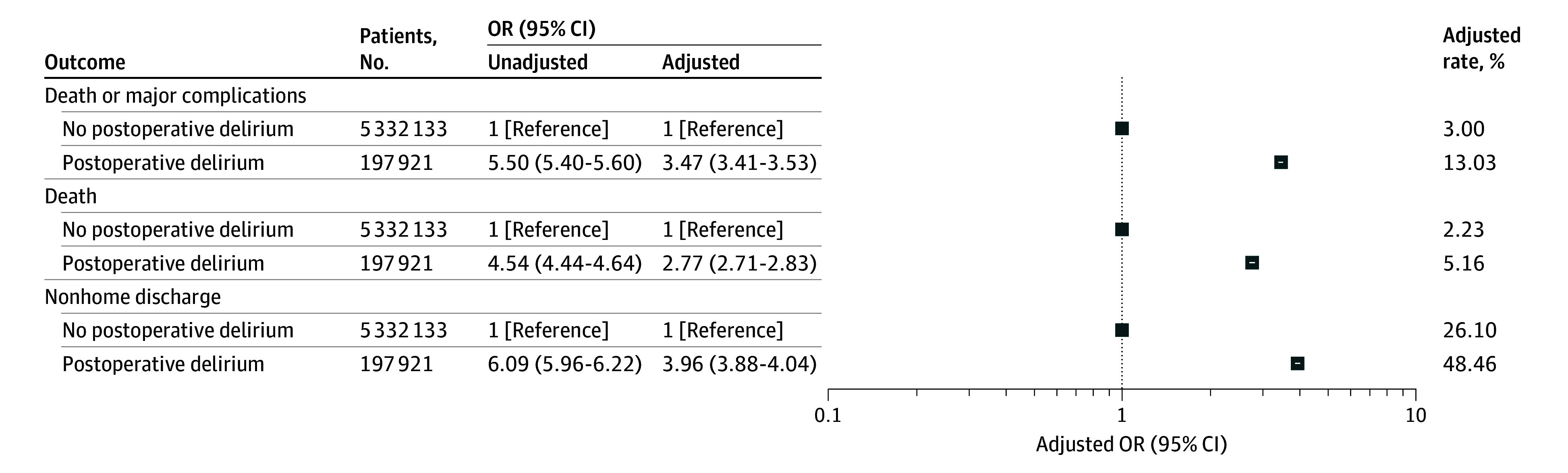
Association of Postoperative Delirium With Death or Major Complications, Death, and Nonhome Discharge Adjusted odds ratios (ORs) were adjusted for patient demographics, payer status; urgency; admission source; frailty; comorbidities; surgical procedure; and day of the week, month, and year of admission. Unadjusted ORs were only adjusted for age and surgery. ORs greater than 1 represent increased postoperative delirium.

The observed incidence of death or major complications was 19.5% (38 585 individuals) in patients with postoperative delirium vs 2.9% (156 309 individuals) in those without postoperative delirium (OR, 5.50; 95% CI, 5.40-5.60; *P* < .001). Similarly, the risk of 30-day mortality (26 148 individuals [13.2%] vs 111 395 individuals [2.1%]; OR, 4.54; 95% CI, 4.44-4.64; *P* < .001) and nonhome discharge (135 948 individuals [68.7%] vs 1 35 980 individuals [24.9%]; OR, 6.09; 95% CI, 5.96-6.22; *P* < .001) were higher in patients with postoperative delirium compared with those without postoperative delirium (Table and Figure 2).

### Adjusted Outcomes

After risk adjustment, postoperative delirium was still associated with major adverse postoperative outcomes. Patients who experienced postoperative delirium had higher odds of death or major complications (adjusted OR [aOR], 3.47; 95% CI, 3.41-3.53; *P* < .001), 30-day mortality (aOR, 2.77; 95% CI,2.71-2.83; *P* < .001), and nonhome discharge (aOR, 3.96; 95% CI, 3.88-4.04; *P* < .001) (Figure 2). These findings were similar in the additional analyses in which we (1) adjusted for hospital characteristics and (2) excluded patients admitted in 2020. In a post hoc analysis, we did not find differences across males and females in the association of death and major complications with postoperative delirium (aOR, 1.01; 95% CI, 0.98-1.04; *P* = .46).

### Hospital Variation in the Adjusted Rate of Postoperative Delirium

The median (IQR) hospital rate of postoperative delirium was 3.02% (1.54%-4.65%). Model details for the nonhierarchical and hierarchical models are presented in eTable 3 in Supplement 1.

**Figure 3.**
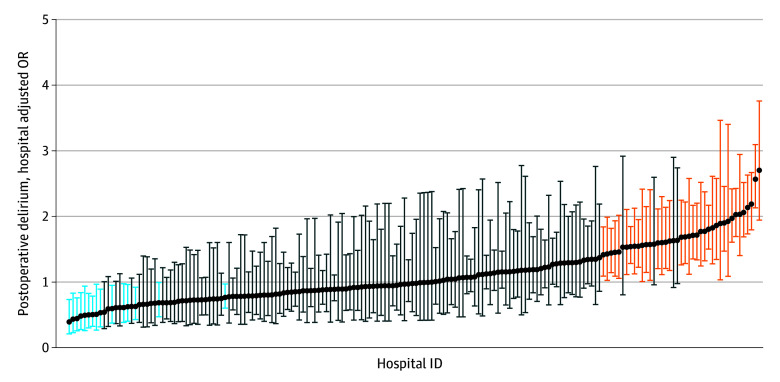
Hospital Risk-Adjusted Odds of Postoperative Delirium Five percent of hospitals in the analytic sample are shown. Vertical bars represent the 95% CIs of the odds ratios (ORs). Hospitals whose performance was below average are shown in orange. Hospitals whose performance was above average are shown in blue. All other hospitals are shown in black. ID indicates identifier.

**Figure 4.**
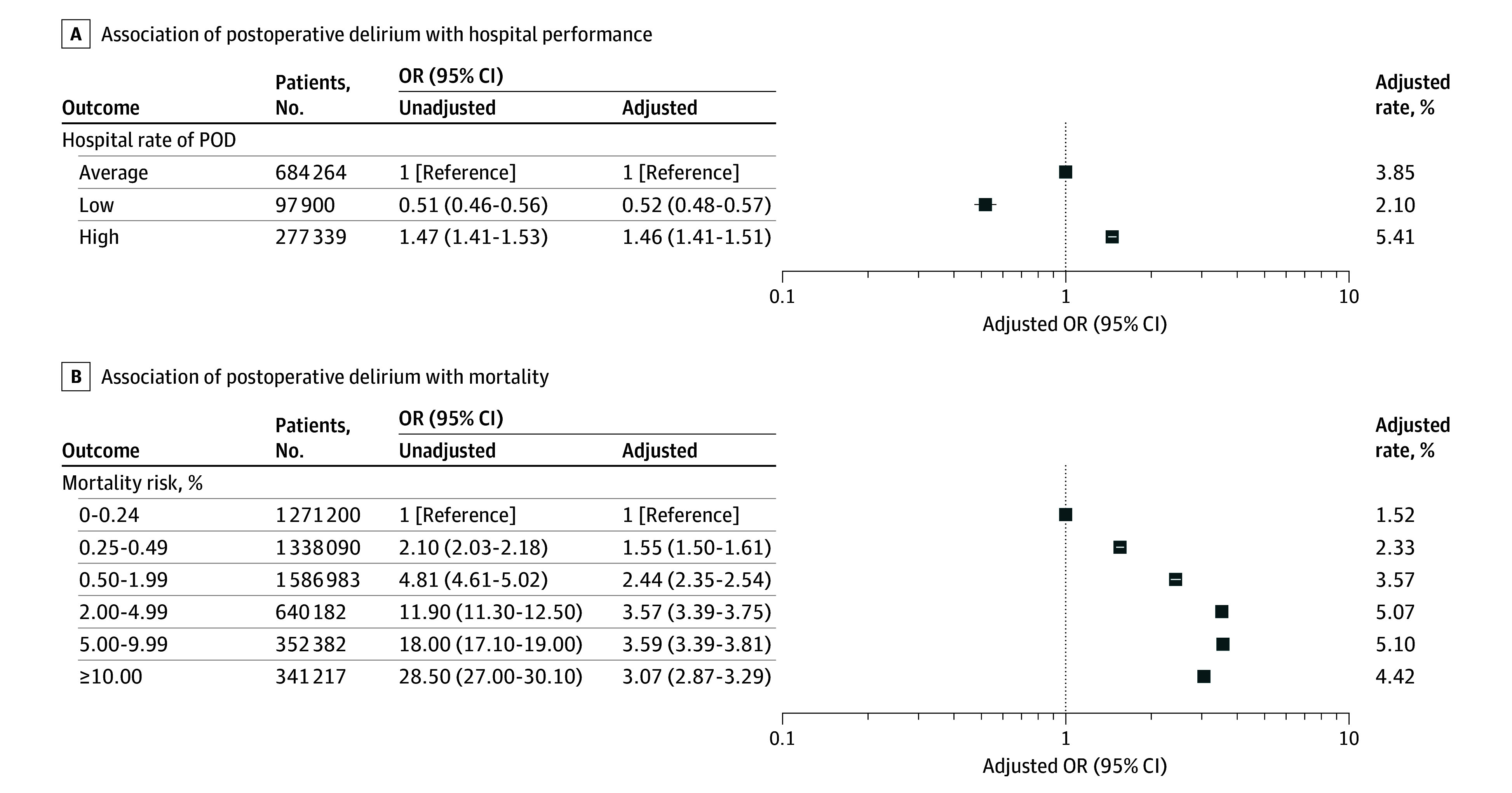
Postoperative Delirium (POD), Hospital Performance, and Risk of Mortality Hospitals with statistically high or lower rates of POD are those hospitals whose risk-adjusted odds ratios (ORs), based on the random-intercept model, were significantly greater or lower than 1. Adjusted ORs were adjusted for patient demographics; payer status; urgency; admission source; frailty; comorbidities; surgical procedure; and day of the week, and month, and year of admission. Unadjusted ORs were only adjusted for age and surgery. ORs greater than 1 represent increased risk of POD.

There was significant variation in the odds of postoperative delirium between hospitals. The variability in the risk-adjusted odds of postoperative delirium is shown in Figure 3. The median OR (1.53; 95% CI, 1.50-1.56) indicates that for 2 patients with similar characteristics treated in different hospitals, the odds of postoperative delirium were 1.5-fold higher in the higher-incidence hospital compared with the lower-incidence hospital. Patients undergoing surgery in 2020 in hospitals identified as having significantly higher risk-adjusted odds of postoperative delirium using 2019 data had higher odds of experiencing postoperative delirium (aOR, 1.46; 95% CI, 1.41-1.51; *P* < .001) compared with hospitals with average odds of postoperative delirium (Figure 4). In contrast, patients undergoing surgery in 2020 in hospitals identified as having significantly lower risk-adjusted odds of postoperative delirium using 2019 data had lower odds of experiencing postoperative delirium (aOR, 0.52; 95% CI, 0.48-0.57; *P* < .001) compared with hospitals with average rates of postoperative delirium (Figure 4).

### Estimated Risk of Mortality and Postoperative Delirium

The odds of postoperative delirium increased in a monotonic fashion as the estimated risk of 30-day mortality increased (Figure 4). Adjusting for age and the surgical procedure, the odds of postoperative delirium increased as the risk of mortality increased from 0.25% to 0.49% (OR, 2.10; 95% CI, 2.03-2.18; *P* < .001), 0.50% to 1.99% (OR, 4.81; 95% CI, 4.61-5.02; *P* < .001), 2.00% to 4.99% (OR, 11.90; 95% CI, 11.30-12.50; *P* < .001), 5.00% to 9.99% (OR, 18.00; 95% CI, 17.10-19.00; *P* < .001), and 10.00% or greater (OR, 28.50; 95% CI, 27.00-30.10; *P* < .001) compared with patients with an estimated risk of mortality less than 0.25%.

## Discussion

In this cohort study using national Medicare data on more than 5.5 million patients undergoing noncardiac surgery, we found that approximately 1 in 30 patients had postoperative delirium, and these individuals had a 5.5-fold higher risk of death or major complications, 4.5-fold higher risk of 30-day mortality, and a 6.1-fold higher risk of nonhome discharge compared with patients without postoperative delirium. These changes persisted after risk adjustment. We also found significant variation in the rate of postoperative delirium across 3169 US hospitals. There was a 2.8-fold difference in the odds of postoperative delirium across hospitals after accounting for differences in patient risk. To our knowledge, our study is the largest study to date to examine the clinical implications of postoperative delirium and the first to report hospital-level variation in this outcome using national population-based Medicare claims data. Together, these findings suggest that postoperative delirium may be an important and potentially modifiable target for hospital quality improvement.

Our first major finding was that postoperative delirium was common among Medicare beneficiaries, occurring between 3% to 4% percent of surgical patients. In our study, we found that advanced age, poor functional status (ie, history of falls), malnutrition, anemia, neuropsychiatric conditions (alcohol misuse, psychoses, and depression), kidney disease, liver dysfunction, cerebrovascular disease, and dementia were associated with postoperative delirium. Precipitating factors, such as surgical invasiveness (open vs percutaneous or laparoscopic procedure), and admission urgency were also associated with postoperative delirium. As the population ages and the nonmodifiable factors outlined previously continue to increase in prevalence, postoperative delirium will be a growing issue among hospitalized patients.

Prior studies suggest that delirium may be preventable in up to 40% of cases.^38^ Our study did not examine the association of potentially reversible factors such as sedative hypnotics, hemodynamic management, depth of anesthesia, or choice of anesthetic technique (ie, regional vs general anesthesia) with postoperative delirium; these are key areas for ongoing research. For example, intraoperative blood pressure management, anesthetic depth, use of regional anesthesia, and choice of anesthetic agents may impact perioperative brain health. A large retrospective study of more than 300 000 patients undergoing noncardiac surgery showed that a mean arterial pressure less than 55 mm Hg was associated with postoperative delirium.^6^ Anesthesia dose is another potentially modifiable contributor, but randomized clinical trials examining the association of anesthesia dose with delirium have reported mixed results. The single-center Electroencephalographic Guidance of Anesthesia to Alleviate Geriatric Syndromes (ENGAGES) trial (Wildes et al^39^) and multicenter ENGAGES-Canada trial by (Deschamps et al^40^) found no differences in the incidence of delirium between an electroencephalogram-guided strategy and usual care.^39,40^ In contrast, a substudy of a multicenter trial^41^ found that postoperative delirium was reduced from 28% to 19% in patients randomized to lighter anesthesia compared with deeper anesthesia. It is also notable and unexpected that the use of regional anesthesia vs general anesthesia did not reduce the incidence of postoperative delirium in 2 large multicenter randomized clinical trials: Regional Versus General Anesthesia for Promoting Independence After Hip Fracture (REGAIN) trial in the US (Neuman et al^42^) and the Regional Anesthesia vs General Anesthesia (RAGA) trial (Li et al^13^) in China. There are, however, nonpharmacologic interventions that are effective for the prevention and treatment of postoperative delirium, such as early mobilization, sleep enhancement, return of hearing aids and eyeglasses, and minimization of restraint use.^43^

Our second major finding was that postoperative delirium was associated with markedly worse short-term outcomes among surgical patients. Most importantly, delirium may lead to long-term cognitive impairment and dementia. A recent meta-analysis of 24 studies^44^ reported that delirium is associated with a 2.3-fold higher risk of cognitive impairment at 3 months or more. A study by Inouye et al^23^ recently reported that delirium was associated with an 8.8-fold higher odds of a new dementia diagnosis. It remains unknown, however, if there is a causal relationship between delirium and long-term cognitive decline and dementia. It is likely that postoperative delirium is both a marker of physiologic vulnerability and contributes to the development of long-term cognitive decline.^23^

Finally, our third major finding using national Medicare data on more than 5.5 million patients was that there was a marked variation in the incidence of postoperative delirium across hospitals. Our findings are consistent with a smaller study based on data on 20 212 patients from the ACS geriatric surgery pilot showing 8.5-fold variation in hospital rates of postoperative delirium.^30^ The results of our population-based study may lead the Centers for Medicare & Medicaid to eventually consider postoperative delirium as an appropriate target for performance measurement as part of its new emphasis on age-friendly hospital measures.^45^ However, efforts to improve accuracy of coding practices of postoperative delirium are necessary before this measure of postoperative brain outcomes can be incorporated into quality reporting and value-based purchasing.

### Limitations

Our study has several important limitations. First and most importantly, the incidence of postoperative delirium is known to be underreported in Medicare claims data. Major randomized clinical trials have reported an incidence between 18% and 26% of postoperative delirium in patients undergoing major surgery.^39,40,41^ The incidence of postoperative delirium was 10.5% in the ACS National Surgical Quality Improvement Program geriatric surgery pilot project.^14^ The reported incidence of postoperative delirium is substantially lower in studies based on administrative data, ranging between 0.8% and 3.7%.^6,8,11,46,47,48^ In our study, the incidence of postoperative delirium, defined using delirium and encephalopathy codes, was 3.6%. We included *ICD-10* diagnostic codes for postoperative delirium and encephalopathy because “delirium and acute encephalopathy describe complementary aspects of a shared set of acute neurocognitive syndromes,”^31^ and hospitals are financially incentivized to code delirium as encephalopathy.^49^ It may be possible that while Medicare claims data undercode postoperative delirium, we are likely identifying more severe and clinically important cases of postoperative delirium, given the magnitude of the association of postoperative delirium with 30-day mortality we observed.

Second, our study does not allow us to make causal inferences regarding the association of postoperative delirium with mortality, complications, and nonhome discharges. In some cases, it is likely that postoperative delirium occurs after a complication instead of before the complication (ie, some patients may develop delirium after heart failure or stroke), thus causing the association of delirium with our composite outcome to be upwardly biased. Notably, the importance of this limitation is offset by the fact that our findings were similar when we examined the association of postoperative delirium with 30-day mortality alone. Third, our study may be biased by unmeasured confounding. This concern is mitigated by our use of comprehensive risk adjustment. Fourth, it is possible that hospital variability in the rate of postoperative delirium may be due to differences in coding accuracy and delirium screening, instead of reflecting true differences in performance. Fifth, the magnitude of the association of postoperative delirium with other important outcomes, such as 30-day mortality, may be biased upwards due to unmeasured confounding despite the use of comprehensive risk adjustment.

## Conclusions

Postoperative delirium is a substantial and preventable complication for older adults under major noncardiac surgery. Using national data on more than 5.5 million noncardiac surgical procedures in patients aged 65 years and older, this retrospective cohort study found that postoperative delirium was associated with a 3.5-fold higher risk of death or major complications, 2.8-fold higher risk of 30-day mortality, and 4.0-fold higher risk of nonhome discharge. We also found a nearly 3-fold variation in the rates of postoperative delirium across US hospitals. The wide variation across hospitals and its association with increased risks of mortality, major complications, and nonhome discharge underscores the need for targeted interventions aimed at improving perioperative brain health.

## Notes

Supplement 1. **eTable 1.** *ICD-10-CM* and *ICD-10*-PCS Codes Used to Identify Patient Characteristics **eTable 2.** Hospital Characteristics **eTable 3.** Models for Postoperative Delirium

Supplement 2.
Data Sharing Statement
