# Supplementary material for: Postoperative Delirium in Older Adults Undergoing Noncardiac Surgery
Source: JAMA Netw Open. 2025 Jul 8;8(7):e2519467. doi: 10.1001/jamanetworkopen.2025.19467 (PMC12238904; doi:10.1001/jamanetworkopen.2025.19467)
Supplement: Supplement 1. — eTable 1. ICD-10-CM and ICD-10-PCS Codes Used to Identify Patient Characteristics eTable 2. Hospital Characteristics eTable 3. Models for Postoperative Delirium [file jamanetwopen-e2519467-s001.pdf]

Supplemental Online Content

Lander HL, Dick AW, Joynt Maddox KE, et al. Postoperative delirium in older adults undergoing noncardiac surgery. *JAMA Netw. Open*. 2025;8(7):e2519467. doi:10.1001/jamanetworkopen.2025.19467

eTable 1. *ICD-10-CM* and *ICD-10-PCS* Codes Used to Identify Patient Characteristics

eTable 2. Hospital Characteristics

eTable 3. Models for Postoperative Delirium

This supplemental material has been provided by the authors to give readers additional information about their work.

© 2025 Lander HL et al. *JAMA Network Open*.

Supplement Table 1. ICD-10-CM and ICD-10-PCS codes used to identifiy patient characteristics.

**Outcomes**	
**Postoperative Delirium**	
Delirium	F13131; F13231; F13931; F19131; 19231; F19931; 19921; R400; R401; R402; R4182;
Encephalopathy	G92; G940; G9341; G3349
**Major Complications**	
Acute myocardial Infarction	I214, I222; I210, I220; I211, I221; I212, I228; I213, I229; I213, I229
Acute ischemic stroke	I63
Congestive heart failure	I50
**Functional Status/frailty**	
Wheelchair use	Z993
Supplemental oxygen	Z9981
Malnutrition	E43; E44; E45; E46
Urinary incontinence	R32
Fecal incontinence	R15
Gait disturbane	R262; R268
History of falls	W00; W01; W02; WO3; W04; W05;W06; W07; W08; W09; W10; W11; W12; W13; W14;W15; W17; W18; W19
Dependent on caregiver	Z74
**Pre-existing Conditions**	
Elxihauser comorbidities	https://hcup-us.ahrq.gov/toolssoftware/comorbidityicd10/comorbidity_icd10.jsp
Myocardial infarction	
Prior ST-segment elevation MI	I210; I220; I211; I221; I212; I228; I213; I229
Prior Non-ST-segment elevation MI	I214; I222
Prior other MI	I219; I21A
COVID-19	U071
CABG	0210; 0211; 0212; 0213

© 2025 Lander HL et al. *JAMA Network Open*.

Supplement Table 2. Hospital Characteristics	
Characteristics	Hospitals, No. (%)
Disproportionate share percentage (DSH)	
0-9.9%	285 (9)
10.0-24.9%	1,024 (32.3)
25.0-49.9%	1,498 (47.3)
50.0%-	362 (11.4)
Resident-to-bed ratio	
0	2,032 (64.1)
>0-0.10	523 (16.5)
0.11-0.20	206 (6.5)
0.21-0.40	217 (6.9)
0.41-	191 (6)
Average daily census	
<20	697 (22)
21-50	599 (18.9)
51-150	1,043 (32.9)
151-300	547 (17.3)
301-	283 (8.9)
Rurality	
Rural hospital	716 (22.6)
Large urban hospital	1,259 (39.7)
Other urban hospital	1,194 (37.7)
Region	
New England	128 (4)
Middle Atlantic	353 (11.1)
South Atlantic	552 (17.4)
East North Central	488 (15.4)
East South Central	260 (8.2)
West North Central	244 (7.7)
West South Central	483 (15.2)
Mountain	223 (7)
Pacific	390 (12.3)
Puerto Rico	48 (1.5)

© 2025 Lander HL et al. *JAMA Network Open*.

Supplement Table 3. Postoperative Delirium.								
	**Non-hierarchical models** (2017-2019 data)						**Hierarchical model** (2019 data)	
	Unadusted		Patient Charactistics		Patient and Hospital Characteristics		Patient Charactistics	
	OR (95% CI)	P	OR (95% CI)	P	OR (95% CI)	P	OR (95% CI)	P
Age								
65-69	reference		reference		reference		reference	
70-74	1.27 (1.25 ,1.29)	0.000	1.32 (1.3 ,1.34)	0.000	1.32 (1.3 ,1.34)	0.000	1.31 (1.28 ,1.36)	0.000
75-79	1.82 (1.79 ,1.86)	0.000	1.76 (1.73 ,1.79)	0.000	1.77 (1.74 ,1.8)	0.000	1.76 (1.71 ,1.82)	0.000
80-84	2.53 (2.48 ,2.59)	0.000	2.17 (2.13 ,2.22)	0.000	2.18 (2.14 ,2.23)	0.000	2.2 (2.13 ,2.27)	0.000
85-89	3.51 (3.43 ,3.6)	0.000	2.55 (2.49 ,2.62)	0.000	2.56 (2.5 ,2.63)	0.000	2.55 (2.46 ,2.65)	0.000
90-94	4.55 (4.42 ,4.69)	0.000	2.82 (2.73 ,2.9)	0.000	2.83 (2.75 ,2.91)	0.000	2.89 (2.76 ,3.02)	0.000
95-	5.72 (5.5 ,5.96)	0.000	3.19 (3.07 ,3.32)	0.000	3.21 (3.08 ,3.34)	0.000	3.34 (3.12 ,3.57)	0.000
Sex								
Male			reference		reference		reference	
Female			0.88 (0.87 ,0.89)	0.000	0.88 (0.87 ,0.89)	0.000	0.89 (0.88 ,0.91)	0.000
Race and Ethnicity								
American Indian and Alaska native			1.01 (0.93 ,1.11)	0.795	1.05 (0.96 ,1.15)	0.33	0.93 (0.81 ,1.08)	0.34
Asian and Pacific Islander			0.81 (0.76 ,0.86)	0.000	0.8 (0.76 ,0.84)	0.000	0.82 (0.76 ,0.88)	0.000
Black			0.89 (0.86 ,0.92)	0.000	0.88 (0.86 ,0.91)	0.000	0.9 (0.86 ,0.93)	0.000
Hispanic			0.73 (0.69 ,0.76)	0.000	0.79 (0.76 ,0.82)	0.000	0.83 (0.79 ,0.87)	0.000
White			reference		reference		reference	
Other			0.93 (0.87 ,0.99)	0.023	0.92 (0.86 ,0.98)	0.006	0.89 (0.79 ,1)	0.06
Dual-eligible			1.08 (1.06 ,1.1)	0.000	1.07 (1.05 ,1.09)	0.000		
Admission urgency								
Elective			reference		reference		reference	
Emergent			1.84 (1.79 ,1.88)	0.000	1.86 (1.82 ,1.9)	0.000	1.97 (1.92 ,2.02)	0.000
Urgent			1.56 (1.51 ,1.62)	0.000	1.59 (1.54 ,1.65)	0.000	1.64 (1.58 ,1.71)	0.000
Admission source								
Community			reference		reference		reference	
Hospital			1.36 (1.32 ,1.41)	0.000	1.27 (1.23 ,1.31)	0.000	1.3 (1.24 ,1.35)	0.000
SNF/Nursing Home			0.98 (0.93 ,1.02)	0.307	0.96 (0.92 ,1.01)	0.093	1.01 (0.94 ,1.08)	0.77
Other			1.16 (1.09 ,1.23)	0.000	1.14 (1.08 ,1.22)	0.000	1.25 (1.13 ,1.37)	0.000

© 2025 Lander HL et al. *JAMA Network Open*.

Supplement Table 3. Postoperative Delirium.								
	**Non-hierarchical models** (2017-2019 data)						**Hierarchical model** (2019 data)	
	Unadusted		Patient Charactistics		Patient and Hospital Characteristics		Patient Charactistics	
	OR (95% CI)	P	OR (95% CI)	P	OR (95% CI)	P	OR (95% CI)	P
Functional Status/frailty								
Wheelchhair use			1.08 (1.01 ,1.15)	0.02	1.06 (0.99 ,1.13)	0.08	1.02 (0.92 ,1.14)	0.69
Supplemental oxygen			1.04 (1.01 ,1.08)	0.01	1.05 (1.01 ,1.08)	0.01	1.07 (1.01 ,1.14)	0.02
Malnutrition			2.19 (2.14 ,2.24)	0.000	2.17 (2.12 ,2.21)	0.000	2.2 (2.14 ,2.27)	0.000
Urinary incontence			1.41 (1.36 ,1.46)	0.000	1.39 (1.34 ,1.44)	0.000	1.38 (1.3 ,1.47)	0.000
Fecal incontinence			1.11 (1.04 ,1.2)	0.004	1.09 (1.01 ,1.17)	0.03	1.07 (0.93 ,1.22)	0.36
Gait disturbance			1.28 (1.21 ,1.36)	0.000	1.3 (1.23 ,1.37)	0.000	1.19 (1.09 ,1.31)	0.000
History of falls			1.35 (1.17 ,1.55)	0.000	1.35 (1.19 ,1.53)	0.000	1.22 (1.03 ,1.45)	0.02
Dependent on caregiver			0.87 (0.78 ,0.97)	0.01	0.9 (0.82 ,0.99)	0.04	0.89 (0.79 ,1.01)	0.06
Previous myocardial infarction								
None			reference		reference		reference	
Prior ST segment elevation MI			2.33 (1.98 ,2.73)	0.000	2.33 (1.99 ,2.74)	0.000	2.4 (1.75 ,3.3)	0.000
Prior Non-ST segment elevation MI			1.05 (0.97 ,1.13)	0.20	1.07 (0.99 ,1.15)	0.10	1.08 (0.93 ,1.26)	0.33
Prior other MI			1.28 (1.19 ,1.38)	0.000	1.26 (1.18 ,1.35)	0.000	1.29 (1.15 ,1.45)	0.000
Prior Coronary Artery Bypass Graft Surgery			0.93 (0.88 ,0.99)	0.022	0.94 (0.88 ,0.99)	0.03	0.97 (0.87 ,1.08)	0.53
COVID-19			1.16 (1.02 ,1.32)	0.025	1.16 (1.02 ,1.31)	0.03		
Elixhauser comorbidities								
AIDS			1.09 (0.96 ,1.24)	0.189	1.08 (0.95 ,1.22)	0.25	1.26 (1 ,1.59)	0.05
Alcohol abuse			2.64 (2.56 ,2.73)	0.000	2.64 (2.56 ,2.73)	0.000	2.65 (2.52 ,2.79)	0.000
Deficiency anemia			0.95 (0.94 ,0.97)	0.000	0.96 (0.95 ,0.98)	0.000	0.99 (0.96 ,1.01)	0.34
Autoimmune conditions			1.1 (1.08 ,1.13)	0.000	1.09 (1.07 ,1.12)	0.000	1.09 (1.04 ,1.14)	0.000
Lymphoma			1.16 (1.11 ,1.22)	0.000	1.14 (1.09 ,1.2)	0.000	1.22 (1.11 ,1.33)	0.000
Leukemia			1.13 (1.07 ,1.2)	0.000	1.12 (1.06 ,1.19)	0.000	1.11 (1 ,1.24)	0.06
Metastatic cancer			1.14 (1.11 ,1.17)	0.000	1.11 (1.09 ,1.14)	0.000	1.09 (1.04 ,1.14)	0.000
Solid tumor			0.98 (0.96 ,1.01)	0.20	0.98 (0.95 ,1)	0.08	0.95 (0.9 ,1)	0.06
Cerebrovascular disease			1.3 (1.26 ,1.33)	0.000	1.3 (1.26 ,1.33)	0.000	1.28 (1.22 ,1.34)	0.000
Heart failure			1.32 (1.29 ,1.34)	0.000	1.31 (1.28 ,1.33)	0.000	1.35 (1.31 ,1.39)	0.000
Coagulopathy			1.27 (1.25 ,1.3)	0.000	1.27 (1.24 ,1.3)	0.000	1.33 (1.27 ,1.38)	0.000
Dementia			2.15 (2.11 ,2.19)	0.000	2.16 (2.12 ,2.21)	0.000	2.28 (2.22 ,2.35)	0.000
Depression			1.4 (1.37 ,1.42)	0.000	1.38 (1.36 ,1.41)	0.000	1.39 (1.36 ,1.43)	0.000
Diabetes without chronic complications			0.95 (0.94 ,0.97)	0.000	0.96 (0.95 ,0.98)	0.000	0.98 (0.95 ,1.01)	0.152
Diabetes with chronic complications			1.4 (1.38 ,1.42)	0.000	1.4 (1.38 ,1.42)	0.000	1.42 (1.38 ,1.45)	0.000
Drug abuse			1.69 (1.6 ,1.77)	0.000	1.68 (1.6 ,1.76)	0.000	1.72 (1.59 ,1.86)	0.000

© 2025 Lander HL et al. *JAMA Network Open*.

**Supplement Table 3. Postoperative Delirium.**								
	**Non-hierarchical models** (2017-2019 data)						**Hierarchical model** (2019 data)	
	Unadusted		Patient Charactistics		Patient and Hospital Characteristics		Patient Charactistics	
	OR (95% CI)	P	OR (95% CI)	P	OR (95% CI)	P	OR (95% CI)	P
Hypertension, complicated			1.25 (1.23 ,1.28)	0.000	1.26 (1.23 ,1.28)	0.000	1.25 (1.2 ,1.29)	0.000
Hypertension, uncomplicated			1 (0.99 ,1.02)	0.61	1 (0.99 ,1.02)	0.58	0.98 (0.96 ,1.01)	0.21
Liver disease, mild			1 (0.97 ,1.03)	0.96	0.99 (0.96 ,1.02)	0.59	0.99 (0.94 ,1.04)	0.57
Liver disease and failure, moderate to severe			1.49 (1.41 ,1.58)	0.000	1.46 (1.38 ,1.54)	0.000	1.54 (1.39 ,1.71)	0.000
Chronic pulmonary disease			1.17 (1.15 ,1.18)	0.000	1.17 (1.16 ,1.18)	0.000	1.17 (1.14 ,1.2)	0.000
Neurologic disorder, movement disorder			1.38 (1.35 ,1.42)	0.000	1.38 (1.35 ,1.41)	0.000	1.37 (1.32 ,1.43)	0.000
Seizures and epilepsy			1.37 (1.33 ,1.41)	0.000	1.37 (1.33 ,1.42)	0.000	1.41 (1.33 ,1.5)	0.000
Obesity			1.05 (1.03 ,1.06)	0.000	1.04 (1.02 ,1.06)	0.000	1.01 (0.99 ,1.04)	0.37
Paralysis			1.19 (1.15 ,1.23)	0.000	1.18 (1.14 ,1.22)	0.000	1.16 (1.1 ,1.22)	0.000
Peripheral vascular disease			1.12 (1.1 ,1.15)	0.000	1.12 (1.1 ,1.15)	0.000	1.16 (1.12 ,1.2)	0.000
Pyschoses			1.51 (1.46 ,1.57)	0.000	1.51 (1.46 ,1.56)	0.000	1.5 (1.41 ,1.58)	0.000
Pulmonary circulation disease			1.19 (1.16 ,1.22)	0.000	1.19 (1.16 ,1.22)	0.000	1.18 (1.13 ,1.24)	0.000
Renal failure, moderate			1.05 (1.03 ,1.07)	0.000	1.04 (1.02 ,1.06)	0.000	1.03 (1 ,1.07)	0.047
Renal failure, severe			1.63 (1.59 ,1.67)	0.000	1.62 (1.58 ,1.66)	0.000	1.59 (1.51 ,1.66)	0.000
Hypothyrodism			0.98 (0.97 ,1)	0.009	0.98 (0.97 ,1)	0.01	0.98 (0.96 ,1)	0.12
Thyroid, other disorders			1.01 (0.97 ,1.06)	0.54	1 (0.96 ,1.05)	0.92	1.02 (0.94 ,1.1)	0.66
Peptic ulcer disease with bleeding			1.12 (1.07 ,1.16)	0.000	1.12 (1.08 ,1.16)	0.000	1.14 (1.06 ,1.23)	0.001
Valvular disease			1.04 (1.02 ,1.06)	0.000	1.04 (1.02 ,1.06)	0.000	1.01 (0.97 ,1.04)	0.67
Procedural approach								
Open	reference		reference		reference		reference	
Percutaneous or laparascopic	0.43 (0.42 ,0.44)	0.000	0.51 (0.5 ,0.53)	0.000	0.51 (0.5 ,0.52)	0.000	0.54 (0.52 ,0.56)	0.000
Procedure								
Abdominal aortic aneurysm repair	9.85 (9.04 ,10.74)	0.000	4.84 (4.43 ,5.28)	0.000	4.45 (4.09 ,4.85)	0.000	4.2 (3.56 ,4.96)	0.000
Bile duct, liver, or pancreatic surgery	5.39 (5.08 ,5.72)	0.000	3.23 (3.04 ,3.43)	0.000	2.94 (2.76 ,3.12)	0.000	2.95 (2.74 ,3.17)	0.000
Carotid endarterectomy	0.96 (0.91 ,1.02)	0.163	0.66 (0.62 ,0.69)	0.000	0.64 (0.6 ,0.67)	0.000	0.66 (0.61 ,0.72)	0.000
Colon surgery	4.05 (3.9 ,4.2)	0.000	2.01 (1.94 ,2.08)	0.000	1.97 (1.9 ,2.04)	0.000	1.95 (1.87 ,2.04)	0.000
Spinal fusion	3.2 (3.06 ,3.34)	0.000	2.48 (2.38 ,2.58)	0.000	2.39 (2.3 ,2.48)	0.000	2.39 (2.3 ,2.49)	0.000
Gastric surgery	4.78 (4.55 ,5.02)	0.000	1.88 (1.79 ,1.98)	0.000	1.86 (1.77 ,1.95)	0.000	1.85 (1.72 ,1.99)	0.000
Hysterectomy	1.74 (1.61 ,1.88)	0.000	1.54 (1.43 ,1.66)	0.000	1.45 (1.35 ,1.56)	0.000	1.44 (1.27 ,1.62)	0.000
Rectal surgery	2.59 (2.42 ,2.76)	0.000	1.64 (1.54 ,1.75)	0.000	1.58 (1.48 ,1.68)	0.000	1.52 (1.36 ,1.72)	0.000

© 2025 Lander HL et al. *JAMA Network Open*.

Supplement Table 3. Postoperative Delirium.								
	**Non-hierarchical models** (2017-2019 data)						**Hierarchical model** (2019 data)	
	Unadusted		Patient Charactistics		Patient and Hospital Characteristics		Patient Charactistics	
	OR (95% CI)	P	OR (95% CI)	P	OR (95% CI)	P	OR (95% CI)	P
Arthoplasty of knee	reference		reference		reference		reference	
Arthoplasty of hip	2.12 (2.05 ,2.19)	0.000	1.27 (1.24 ,1.3)	0.000	1.25 (1.22 ,1.28)	0.000	1.18 (1.14 ,1.22)	0.000
Laminectomy	2.39 (2.27 ,2.51)	0.000	1.7 (1.62 ,1.78)	0.000	1.62 (1.55 ,1.7)	0.000	1.57 (1.48 ,1.67)	0.000
Neck surgery	2.03 (1.84 ,2.25)	0.000	1.51 (1.36 ,1.66)	0.000	1.34 (1.21 ,1.48)	0.000	1.23 (1.04 ,1.45)	0.02
Kidney surgery	3.07 (2.9 ,3.25)	0.000	2.26 (2.14 ,2.39)	0.000	2.13 (2.02 ,2.25)	0.000	2.16 (1.99 ,2.35)	0.000
Ovarian surgery	2.21 (2 ,2.46)	0.000	1.82 (1.64 ,2.01)	0.000	1.68 (1.52 ,1.86)	0.000	1.72 (1.42 ,2.07)	0.000
Prostate surgery	1.2 (1.1 ,1.32)	0.000	1.05 (0.96 ,1.15)	0.25	1.01 (0.92 ,1.1)	0.90	1.04 (0.88 ,1.22)	0.65
Peripheral vascular bypass surgery	3.33 (3.16 ,3.51)	0.000	1.64 (1.56 ,1.72)	0.000	1.57 (1.49 ,1.65)	0.000	1.53 (1.42 ,1.64)	0.000
Small bowel surgery	4.6 (4.42 ,4.78)	0.000	1.96 (1.89 ,2.03)	0.000	1.9 (1.83 ,1.97)	0.000	1.91 (1.81 ,2)	0.000
Spleen surgery	5.69 (5.19 ,6.25)	0.000	2.5 (2.28 ,2.74)	0.000	2.36 (2.15 ,2.59)	0.000	2.18 (1.82 ,2.6)	0.000
Thoracic surgery	4.49 (4.29 ,4.7)	0.000	2.32 (2.22 ,2.43)	0.000	2.22 (2.12 ,2.32)	0.000	2.16 (2.05 ,2.29)	0.000
Thyroid and/or parathyroid surgery	1.38 (1.22 ,1.56)	0.000	0.94 (0.84 ,1.06)	0.31	0.89 (0.8 ,0.99)	0.04	0.92 (0.75 ,1.11)	0.37
Exploratory laparotomy	4.18 (4.01 ,4.36)	0.000	1.59 (1.52 ,1.65)	0.000	1.55 (1.49 ,1.61)	0.000	1.47 (1.39 ,1.56)	0.000
Gallbladder surgery	3.2 (3.07 ,3.35)	0.000	1.24 (1.19 ,1.29)	0.000	1.22 (1.17 ,1.27)	0.000	1.13 (1.06 ,1.2)	0.000
Hospital Characteristics								
Disproportionate share percentage (DSH)								
0-9.9%					reference			
10.0-24.9%					1.05 (0.94 ,1.16)	0.39		
25.0-49.9%					1.09 (0.98 ,1.21)	0.10		
50.0%-					0.91 (0.8 ,1.03)	0.14		
Resident-to-bed ratio								
0					reference			
>0-0.10					0.97 (0.93 ,1.02)	0.246		
0.11-0.20					1.03 (0.97 ,1.1)	0.317		
0.21-0.40					1.05 (0.97 ,1.14)	0.210		
0.41-					1.15 (1.07 ,1.24)	0.000		
Average daily census								
<20					reference			
21-50					1.14 (1.03 ,1.26)	0.01		
51-150					1.24 (1.11 ,1.37)	0.000		
151-300					1.26 (1.14 ,1.4)	0.000		
301-					1.39 (1.25 ,1.56)	0.000		

© 2025 Lander HL et al. *JAMA Network Open*.

Supplement Table 3. Postoperative Delirium.								
	**Non-hierarchical models** (2017-2019 data)						**Hierarchical model** (2019 data)	
	Unadusted		Patient Charactistics		Patient and Hospital Characteristics		Patient Charactistics	
	OR (95% CI)	P	OR (95% CI)	P	OR (95% CI)	P	OR (95% CI)	P
Rurality								
Rural hospital					reference			
Large urban hospital					0.95 (0.89 ,1)	0.07		
Other urban hospital					0.91 (0.86 ,0.97)	0.002		
Region								
New England					reference			
Middle Atlantic					0.76 (0.7 ,0.84)	0.000		
South Atlantic					0.83 (0.77 ,0.91)	0.000		
East North Central					0.88 (0.82 ,0.96)	0.002		
East South Central					0.89 (0.8 ,0.98)	0.02		
West North Central					0.95 (0.87 ,1.04)	0.23		
West South Central					0.85 (0.78 ,0.93)	0.000		
Mountain					0.87 (0.79 ,0.95)	0.003		
Pacific					0.91 (0.84 ,0.99)	0.03		
Puerto Rico					0.31 (0.14 ,0.67)	0.003		
Day of admission								
Sunday			reference		reference			
Monday			0.99 (0.96 ,1.01)	0.30	0.99 (0.96 ,1.01)	0.34		
Tuesday			0.9 (0.88 ,0.92)	0.000	0.9 (0.88 ,0.92)	0.000		
Wednesday			0.89 (0.87 ,0.91)	0.000	0.9 (0.88 ,0.92)	0.000		
Thursday			0.91 (0.89 ,0.93)	0.000	0.92 (0.9 ,0.94)	0.000		
Friday			0.93 (0.91 ,0.95)	0.000	0.93 (0.91 ,0.95)	0.000		
Saturday			0.97 (0.94 ,0.99)	0.002	0.96 (0.94 ,0.98)	0.001		
Month of admission								
January			reference		reference			
February			1.01 (0.98 ,1.03)	0.65	1.01 (0.98 ,1.03)	0.59		
March			0.99 (0.97 ,1.01)	0.28	0.99 (0.97 ,1.01)	0.31		
April			1.01 (0.99 ,1.03)	0.36	1.01 (0.99 ,1.03)	0.35		
May			1.01 (0.99 ,1.04)	0.26	1.01 (0.99 ,1.04)	0.26		
June			1 (0.98 ,1.02)	0.96	1 (0.98 ,1.02)	0.98		
July			1.01 (0.99 ,1.03)	0.51	1.01 (0.98 ,1.03)	0.62		
August			1.01 (0.99 ,1.03)	0.42	1.01 (0.99 ,1.03)	0.46		

© 2025 Lander HL et al. *JAMA Network Open*.

Supplement Table 3. Postoperative Delirium.								
	**Non-hierarchical models** (2017-2019 data)						**Hierarchical model** (2019 data)	
	Unadusted		Patient Charactistics		Patient and Hospital Characteristics		Patient Charactistics	
	OR (95% CI)	P	OR (95% CI)	P	OR (95% CI)	P	OR (95% CI)	P
September			1 (0.98 ,1.03)	0.67	1 (0.98 ,1.03)	0.76		
October			1.01 (0.99 ,1.03)	0.38	1.01 (0.99 ,1.03)	0.42		
November			1.02 (1 ,1.04)	0.13	1.02 (0.99 ,1.04)	0.15		
December			1.01 (0.98 ,1.03)	0.53	1.01 (0.98 ,1.03)	0.58		
Year								
2017			reference		reference			
2018			0.98 (0.96 ,0.99)	0.004	0.98 (0.96 ,0.99)	0.004		
2019			0.93 (0.91 ,0.95)	0.000	0.93 (0.91 ,0.95)	0.000		
2020			0.97 (0.95 ,0.99)	0.004	0.97 (0.95 ,0.99)	0.002		

© 2025 Lander HL et al. *JAMA Network Open*.
