# Supplementary material for: Postoperative Delirium in Older Adults Undergoing Noncardiac Surgery
Source: JAMA Netw Open. 2025 Jul 8;8(7):e2519467. doi: 10.1001/jamanetworkopen.2025.19467 (PMC12238904; doi:10.1001/jamanetworkopen.2025.19467)
Supplement: Supplement 2. — Data Sharing Statement [file jamanetwopen-e2519467-s002.pdf]

Data Sharing Statement

Lander. Postoperative Delirium in Older Adults Undergoing Noncardiac Surgery. *JAMA Netw Open*. Published July 08, 2025. doi:10.1001/jamanetworkopen.2025.19467

Data
Data available: No

Additional Information
Explanation for why data not available: CMS data was used in this study and is only available with a DUA from CMS.
